# Fontan Circulation and High Cardiac Output State: A Forgotten Phenotype

**DOI:** 10.1016/j.cjcpc.2024.10.001

**Published:** 2024-10-09

**Authors:** Hilary Bews, Ishika Mittal, James W. Tam, Ashish H. Shah

**Affiliations:** a Section of Cardiology, Department of Internal Medicine, Rady Faculty of Health Sciences, University of Manitoba, Winnipeg, Manitoba, Canada; b Institute of Cardiovascular Sciences, Albrechtsen Research Center, St. Boniface Hospital, Winnipeg, Manitoba, Canada

The establishment of Fontan circulation is aimed at preserving systemic saturation and avoiding volume loading of the available ventricle. However, creation of such a circulation leads to the most commonly observed haemodynamic phenotype of elevated venous (Fontan) pressure and low/preserved cardiac output (CO).^1^ Fontan patients are also known to have a blunted augmentation in CO with exercise.^2^ Others and we have previously demonstrated a high output cardiac state (HOCS; cardiac index [CI] >4 L/min/m^2^) among patients with biventricular circulation that is associated with excess morbidity and mortality.^3^^,^^4^ In contrast to general perception, some Fontan patients may have an abnormally high CO.

At our centre, 15 of 62 (24%) Fontan patients have been investigated by cardiac catheterization, and 4 of 15 individuals were noted to have HOCS. These patients were referred either for routine surveillance or with a concern for failing Fontan circulation.^5^ Three patients were male, and tricuspid atresia was the original congenital diagnosis in 3 subjects. Their demographics, original congenital defect, associated anomalies, and investigational details are described in Table 1.

**Table 1:** Demographics and investigational details of the Fontan patients

Demographics	Patient 1	Patient 2	Patient 3	Patient 4
Age (y)	19	25	23	26
Biological sex	Female	Male	Male	Male
BMI (kg/m^2^)	16.3	25.9	26.7	15.2
Original congenital diagnoses	Tricuspid atresia with hypoplastic right ventricle VSD	Tricuspid atresia with hypoplastic right ventricle TGA Pulmonic stenosis Double outlet LV Myxomatous MV with moderate MR	Hypoplastic left ventricle with severe AS and MS	Tricuspid atresia VSD
Current anatomy	Extracardiac Fontan	Extracardiac Fontan Mild-to-moderate AI	Extracardiac Fontan Repaired coarctation of aorta Moderate TR	Extracardiac Fontan
Cirrhosis	Yes	Yes	Yes	Yes
Fontan fenestration present (Y/N)	Residual leak through a closure device	Yes	No	No
Presence of venovenous collaterals (Y/N)	Yes Venovenous collaterals from a hepatic vein Also, residual leak through previously coiled VV collaterals	Yes Venovenous collaterals from the left innominate vein	Yes Venovenous collaterals from innominate, right, and left hepatic veins	No
Presence of pulmonary AV malformation (PAVM) (Y/N)	No	Yes	No	Yes
Relevant medications	None	ACEi	Pulmonary vasodilators ACEi	None
*Transthoracic echocardiogram*				
Systemic ventricular EF (%)	>60	55-60	45-50	50-55
Stroke volume (mL/beat)	64	98	83	95
AV valve regurgitation (Y/N)	No	Yes	Yes	No
Severity of AVV regurgitation	NA	Moderate	Moderate	NA
*Haemodynamics at the time of cardiac catheterization*				
MAP (mm Hg)	60	97	84	100
Mean RAP (mm Hg)	17	15	22	20
Perfusion pressure (mm Hg)	43	82	62	80
Mean PAP	13	15	18	18
Mean PCWP	11	11	14	8
PVR (Wood unit)	0.31	0.43	0.54	1.56
SVR (dynes/s/cm^−5^)	544	753	669	999
Cardiac output (L/min)	6.36	8.72	7.42	6.4
Cardiac index (L/min/m^2^)	4.58	3.96	4.12	4.39
Pulmonary artery saturation (%)	71.1	78.6	75.9	74.8
Aortic saturation (%)	88.5	90.6	94	87.9
*Blood work*				
Haemoglobin	156	177	166	182
Hct (%)	0.473	0.542	0.49	0.54

ACEi, angiotensin-converting enzyme inhibitor; AI, aortic insufficiency, AS, aortic saturation; ASD, atrial septal defect; AV, atrioventricular; AVV, atrioventricular valve; BMI, body mass index; EF, ejection fraction; Hct, hematocrit; LV, left ventricle; MAP, mean aortic pressure; MR, mitral regurgitation; MS, mitral stenosis; MV, mitral valve; NA, not applicable; PAP, pulmonary artery pressure; PCWP, pulmonary capillary wedge pressure; PVR, pulmonary vascular resistance; RAP, right atrial pressure; SVR, systemic vascular resistance; TGA, transposition of the great arteries; TR, tricuspid regurgitation; VSD, ventricular septal defect; VV, venovenous.

The mean right atrial pressure was elevated between 15 and 22 mm Hg. The mean pulmonary arterial pressure ranged from 13 to 18 mm Hg, and the mean pulmonary capillary wedge pressure was within normal limits between 8 and 14 mm Hg. Using the Fick method, CO ranged between 6.4 and 8.7 L/min (CI: 4.0-4.6 L/min/m^2^). Consistent with the pathophysiology of the HOCS, systemic vascular resistance (SVR) was low, between 544 and 999 dynes/s/cm^−5^. Venous-venous collaterals and pulmonary arteriovenous malformations were common in the cohort. All 4 patients demonstrated evidence of cirrhosis on ultrasound or magnetic resonance imaging, and 1 patient had a liver biopsy demonstrating stage 2-3 fibrosis. The ejection fraction of the systemic ventricle was preserved or mildly impaired. All 4 individuals are alive till date.

Although normal haemodynamic parameters for patients with univentricular circulation have not been defined, high CO has been described and is associated with excess mortality.^6^^,^^7^ In 161 Fontan patients (≥15 years after surgery), a CI ≥3 L/min/m^2^ was associated with increased mortality at 5-year follow-up, with a hazard ratio of 2.5 in comparison with early cohort (0.5-5 years after Fontan completion).^7^ Interestingly, when high CI was associated with high Fontan pressures (≥14 mm Hg), observed mortality was even greater, with a hazard ratio of 18.1.^7^ In a separate study from the Mayo Clinic, mortality was 41% at 5 years in Fontan patients with an elevated CI ≥2.5 L/min/m^2^ and high Fontan pressures (≥15 mm Hg) as compared with 4% in patients with low/normal CI and normal Fontan pressures (≤15 mm Hg).^6^ It is worth noting that the definitions used to describe high CI or higher Fontan pressures were different in both of these studies, whereas we have opted to use the definition commonly used for individuals with normal biventricular circulation. Moreover, elevated Fontan pressure in the presence of higher CO may be representative of stiff left ventricle or limited pulmonary vascular reserve (defined as the mean pulmonary artery pressure/CO slope of >3 mm Hg/L/min).^8^ On the other hand, haemodynamic parameters were compared between 27 symptomatic adult Fontan patients and 54 asymptomatic paediatric Fontan patients.^9^ Central venous pressure (CVP) was higher (18 ± 6 mm Hg vs 14 ± 3 mm Hg), SVR index lower (1680 ± 368 vs 1960 ± 550 dynes/cm^5^/m^2^), and CI relatively preserved (2.7 ± 0.8 vs 2.8 ± 0.7 L/min/m^2^) in the symptomatic adult Fontan patients in comparison with the asymptomatic paediatric cohort,^9^ albeit the cohort size is smaller than the other 2 studies. Liver disease was more advanced in the symptomatic Fontan group, and the authors hypothesized that this may be a driver for the haemodynamic changes seen.^10^

The findings of an elevated CO/CI may be multifactorial and could occur as a result of (1) Fontan-associated liver disease and subsequent splanchnic and peripheral arterial vasodilation, with contributions from portosystemic shunting, bacterial translocation, and autonomic nervous system dysfunction; (2) Fontan fenestration; (3) venovenous collaterals; and/or (4) pulmonary arteriovenous malformations (PAVM).^10^^,^^11^ Although the quantification of blood shunting through these defects remains challenging, the end result is systemic desaturation or hypoxaemia, which leads to further systemic vasodilation.^12^^,^^13^ The predisposition towards PAVM may be due to a lack of the “*hepatic factor*” or a manifestation of hepatopulmonary syndrome that is generally reported in patients with biventricular circulation and liver disease; however, differentiating both of these defects in Fontan patients is challenging. Our patients had evidence of cirrhosis on imaging, and the presence of Fontan fenestration and venovenous collaterals, in keeping with these theories. It is important to note the limitations in CO determination in the Fontan population. Given multiple sources of blood supply to the pulmonary circulation, thermodilution is inaccurate.^10^ Although the Fick method is preferred for CO determination, it can be limited by the use of assumed rather than measured oxygen consumption, difficulties in obtaining accurate mixed venous oxygen saturations, challenges with measuring blood passing through venovenous collaterals, Fontan fenestrations, PAVM, and aortopulmonary collaterals.^10^

Differentiating Fontan patients with the HOCS from those with low CO has important treatment implications. Patients with failing Fontan circulation are typically treated with pulmonary vasodilators, angiotensin-converting enzyme inhibitors/angiotensin receptor blockers, or other standard heart failure therapies,^12^ whereas in patients with the HOCS, such medical therapies are likely to lower the SVR further, resulting in adverse outcomes.^12^ Miike et al.^12^ described the use of vasoconstrictors as a potential treatment in a group of 10 patients with failing Fontan haemodynamics and HOCS. Patients were characterized by a high CVP (median 16 mm Hg), low SVR, high CI (median 4.6 L/min/m^2^), and low oxygen saturation (median SpO_2_ 89%). All 10 patients were treated with vasoconstrictor therapy in the form of a noradrenaline infusion or midodrine. After a median of 1.7 months, the median systolic blood pressure, oxygen saturation, and SVR increased; however, no significant changes were seen in CVP or CI. Overall, at a median of 21-month follow-up, the study population experienced high morbidity and mortality, as evidenced by hepatic encephalopathy in 5 patients (50%) and death in 6 patients (60%). However, the number of hospital readmissions was significantly decreased with the use of vasoconstrictor therapy (from a median of 4 to 1).^12^ Their study, although small, suggests that vasoconstrictor therapy may be a valuable tool for preventing hospitalizations in Fontan patients with HOCS. Liver transplantation offers a curative treatment for hepatopulmonary syndrome; however, current guidelines do not support the same for high output state in the presence of liver pathology. Similarly, patients with failing Fontan circulation are usually referred for heart vs heart-liver transplantation. HOCS or hepatopulmonary syndrome could be another indication favouring heart-liver transplantation.

We have found several Fontan patients with HOCS, a haemodynamic phenotype that appears not to be uncommon. A national registry evaluating all-comer Fontan patients is likely to improve our understanding of such a haemodynamic phenotype and may allow us to identify appropriate therapeutic interventions. The Fontan patients should be investigated by catheterization routinely as well as when clinically indicated for precise haemodynamic characterization.^5^
